# An inflamed necrotic appendix epiploicum with immediate contact to a non-inflamed appendix vermiformis: a case report

**DOI:** 10.1186/1752-1947-3-57

**Published:** 2009-02-10

**Authors:** Michael Sand, Gerd Bonhag, Falk-Georges Bechara, Daniel Sand, Benno Mann

**Affiliations:** 1 Department of General and Visceral Surgery, Augusta Krankenanstalt, Academic Teaching Hospital of the Ruhr-University Bochum, Bergstr 26, 44791 Bochum, Germany; 2 Department of Dermatology and Allergology, Ruhr-University Bochum, Gudrunstr 56, 44791 Bochum, Germany; 3 Department of Physiological Science, University of California Los Angeles (UCLA), 621 Charles E Young Drive South, Los Angeles, CA 90095-1527, USA

## Abstract

**Introduction:**

Epiploic appendagitis is a rare cause of focal abdominal pain which, depending on its localisation, can mimic a variety of abdominal diseases. We describe a patient with an inflamed necrotic appendix epiploicum with immediate contact to a non-inflamed appendix vermiformis mimicking acute appendicitis. Considering the rare localization, this is the first report of this kind in the literature.

**Case presentation:**

We present the case of a 50-year-old Caucasian man who presented with classic signs of acute appendicitis. On clinical exam, McBurney and Blumberg signs were positive. Additionally he had fever, leucocytosis (12/nl) and a slight increase in C-reactive protein (1 mg/dl). Based on the clinical presentation, the patient was taken to the operating room to perform an appendicectomy. Surprisingly, we found an inflamed necrotic appendix epiploicum, located immediately on a non-inflamed appendix vermiformis, which was ligated and excised.

**Conclusion:**

This case report demonstrates that epiploic appendagitis can mimic acute appendicitis on clinical exam and should be considered in the broad spectrum of abdominal disease presenting with right lower quadrant pain.

## Introduction

Appendices epiploicae are 50 to 100 fatty appendages located on the three large muscle bands known as the taenia coli. They are supplied by small end-arteries originating from the colon. In cases of torsion, the blood supply of the appendage is altered and infarction of the appendage can result. This can be etiological for right or left lower abdominal quadrant pain which can mimic diverticulitis, appendicitis and a variety of other abdominal diseases clinically [1].

The taenia coli mostly fuse together to become the appendix vermiformis. In this case report, we describe a patient with an inflamed, necrotic appendix epiploicum lying immediately on a non-irritated appendix vermiformis mimicking acute appendicitis.

## Case presentation

We report on a 50-year-old Caucasian man with a 72-hour history of right lower quadrant pain. Three days before presenting in our department, the pain had started in the mid-epigastrium and had gradually shifted to the right lower quadrant. There were no other signs of abdominal pathology, no nausea or vomiting. Bowel movements and micturition were described as normal. Besides his obesity, he was suffering from hypertension and hypercholesterinaemia. He had a history of previous abdominal surgery because of a stab wound injury 17 years ago.

On clinical exam, the abdomen was markedly tender with rebound tenderness in the right lower abdominal quadrant (positive Blumberg sign) and positive McBurney sign. The remaining abdomen was soft. There was no sensitivity on percussion and psoas sign was negative. Peristalsis was normal in all four abdominal quadrants, the kidneys were non-tender on palpation, the recto-digital exam was normal. Echographic findings showed dilation of intestinal loops and pathognomonic pendulating bowel motion.

Laboratory findings revealed leucocytosis (white blood cell count, 12/nl) and a slightly elevated C-reactive protein (CRP, 1 mg/dl). The patient had begun experiencing fever with the onset of pain 3 days before.

**Figure 1:**
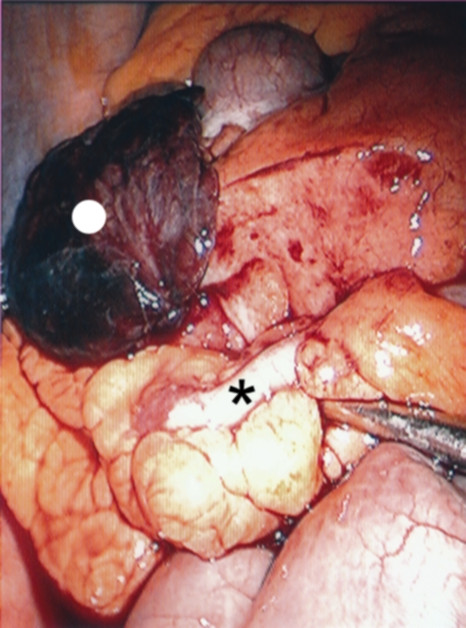
**A raised necrotic, inflamed appendix epiploicum (white spot) which was lying immediately on a non-irritated appendix vermiformis (black star)**.

The patient was clinically diagnosed as having acute appendicitis and was taken to the operating room. At diagnostic laparoscopy, we found a macroscopically normal, non-irritated appendix vermiformis. An inflamed, necrotic appendix epiploicum was found lying immediately above the non-irritated appendix vermiformis causing the patient's symptoms (Figure 1). The necrotic appendix epiploicum was ligated and excised laparoscopically. The postoperative course was uncomplicated and the patient was discharged from the hospital on the second postoperative day.

## Discussion

Inflammation and necrosis of appendices epiploicae is one potential cause of acute abdominal pain and a differential diagnosis for a variety of abdominal diseases. It is a condition which is difficult to diagnose due to the lack of pathognomic clinical features. In addition, it is still a rare diagnosis with low awareness among general surgeons and emergency physicians often causing a diagnostic dilemma. However, there are some characteristics which have been described. Patients are middle aged (40 to 45 years) and present with sharp localized pain mostly in the right or left lower abdominal quadrant [2]. Additional signs of abdominal pathology such as nausea, vomiting or diarrhoea are absent. White blood cell count (WBC) is normal to slightly elevated with a slight increase in CRP values (0.1 to 1.5 mg/dl). Ultrasonography can show a characteristic hyperechoic, non-compressible ovoid mass adjacent to the colonic wall [3]. In doubtful cases, a computed tomography (CT) scan may be used to aid the correct diagnosis [4]. Therapy is widely conservative with oral anti-inflammatory medication, however, if diagnosed intra-operatively, ligation and excision of the necrotic tissue is favoured [5]. In doubtful cases, we prefer exploratory laparoscopy as a curative form of therapy rather than CT scans to avoid radiation exposure in healthy young to middle aged individuals. However, there is a lack of studies dealing with alternative therapy for inflamed appendices epiploicae which is a topic of controversy in the medical literature.

## Conclusion

Inflammation and subsequent necrosis of a pericecal appendix epiploicum should be considered as a differential diagnosis of right lower abdominal quadrant pain potentially mimicking acute appendicitis.

## Consent

Written informed consent was obtained from the patient for publication of this case report and any accompanying images. A copy of the written consent is available for review by the Editor-in-Chief of this journal.

## Competing interests

The authors declare that they have no competing interests.

## Authors' contributions

MS documented and prepared the draft, made substantial contributions to interpretation. GB was the surgeon who performed the operations, made substantial contributions to interpretation and helped in preparing the manuscript. FGB was involved in drafting the manuscript and has made substantial contributions to interpretation. DS was involved in drafting the manuscript and has made contributions to interpretation, performed the literature search, revision of the bibliography and helped with editing of the manuscript. BM edited part of the manuscript and was involved in drafting the manuscript. All authors have read and approved the final version.

## References

[B1] Vinson DR Epiploic appendagitis: a new diagnosis for the emergency physician. Two case reports and a review J Emerg Med 1999 17 827 832 10.1016/S0736-4679(99)00090-6 10499697

[B2] Sand M Gelos M Bechara FG Sand D Wiese TH Steinstraesser L Mann B Epiploic appendagitis – clinical characteristics of an uncommon surgical diagnosis BMC Surg 2007 7 11 10.1186/1471-2482-7-11 17603914 PMC1925058

[B3] Molla E Ripolles T Martinez MJ Rosello E Primary epiploic appendagitis: US and CT findings Eur Radiol 1998 8 435 438 10.1007/s003300050408 9510579

[B4] Rao PM Wittenberg J Lawarason JN Primary epiploic appendagitis: evolutionary changes in CT appearance Radiology 1997 204 713 717 9280248 10.1148/radiology.204.3.9280248

[B5] Singh AK Gervais DA Hahn PF Sagar P Mueller PR Novelline RA Acute epiploic appendagitis and its mimics Radiographics 2005 25 1521 1534 10.1148/rg.256055030 16284132

